# Bone Marrow Adipose Tissue: A New Player in Cancer Metastasis to Bone

**DOI:** 10.3389/fendo.2016.00090

**Published:** 2016-07-14

**Authors:** Emma V. Morris, Claire M. Edwards

**Affiliations:** ^1^Nuffield Department of Surgical Sciences, University of Oxford, Oxford, UK; ^2^Nuffield Department of Orthopaedics, Rheumatology and Musculoskeletal Sciences, University of Oxford, Oxford, UK

**Keywords:** cancer-induced bone disease, marrow adipose tissue, multiple myeloma, prostate cancer, bone metastasis

## Abstract

The bone marrow is a favored site for a number of cancers, including the hematological malignancy multiple myeloma, and metastasis of breast and prostate cancer. This specialized microenvironment is highly supportive, not only for tumor growth and survival but also for the development of an associated destructive cancer-induced bone disease. The interactions between tumor cells, osteoclasts and osteoblasts are well documented. By contrast, despite occupying a significant proportion of the bone marrow, the importance of bone marrow adipose tissue is only just emerging. The ability of bone marrow adipocytes to regulate skeletal biology and hematopoiesis, combined with their metabolic activity, endocrine functions, and proximity to tumor cells means that they are ideally placed to impact both tumor growth and bone disease. This review discusses the recent advances in our understanding of how marrow adipose tissue contributes to bone metastasis and cancer-induced bone disease.

## Introduction

For the majority of cancers, surgical removal of an isolated primary tumor can be curative. However, once tumor cells establish residence in other organs patient mortality is markedly increased. Once detached from the primary site, a single or cluster of tumor cells can circulate the body and establish secondary lesions in distant sites. Metastasis is a highly inefficient process with <0.1% of disseminating cells surviving to form secondary lesions (1). This implies that healthy tissues display a level of hostility toward invading tumor cells, and so in order to survive and repopulate new sites these cells need to have adapted to seek out more permissive environments to occupy. Interestingly, it has long been recognized that each type of tumor has a distinct pattern of dissemination. In 1889, Stephen Paget published a paper proposing that disseminating cancer cells or “seeds” would only colonize secondary sites or “soils” that were compatible with their growth (2). This theory was challenged in the 1920s by James Ewing who proposed that the colonization of secondary sites could be explained purely by circulatory patterns between the primary and secondary sites (3). In fact, these theories are not mutually exclusive, as there is evidence to support both (1). Systemic breast cancer commonly metastasizes to the bones, lungs, liver, and brain most of which do not have a direct circulatory link to breast tissue. Advanced prostate cancer also selectively metastasizes to bone. By contrast, patients suffering from colon cancer often develop initial metastases in the liver (4). Therefore, mechanical factors that govern the amount of cancer cells delivered to an organ, and compatibility factors such as whether the organ preferentially supports the growth of a specific tumor cell type, contribute to cancer spread (5). Once a cancer cell has successfully seeded itself, it is then dependent on the molecular interactions between it and the environment of the new organ. The more permissive the environment, the more likely the tumor cell is able to take advantage and thrive.

## The Bone Microenvironment

Bone or bone marrow is a major target organ for metastasis, providing a fertile “soil” for circulating tumor cells to settle and repopulate. It is the preferential site of a number of solid tumor metastases including breast and prostate, of which 70–80% of advanced cases have bone involvement (6, 7). It is also the site of the fatal hematological malignancy, multiple myeloma. The bone consists of a number of different cell types, including endothelial cells and nerve cells, cells of hematopoietic origin, such as hematopoietic stem cells, osteoclasts macrophages, and lymphocytes, as well as cells from mesenchymal origin, such as chondrocytes, osteoblasts, and adipocytes. Together these cells provide a supportive niche via cellular cross-talk that maintains healthy bone. It is thought that cancer cells are able to hijack this support network to transform what was initially a beneficial niche into a deadly one. Considerable research efforts have been made, attempting to understand the complexity of the bone microenvironment and defining the exact contribution of each cell type in tumor growth and metastasis. Mundy and colleagues greatly broadened our understanding of this by introducing the concept of the “vicious-cycle” involving bi-directional interactions of tumor cells and bone cells, resulting in osteolysis and in turn, tumor growth. Breast cancer cells produce parathyroid hormone-related peptide (PTHrP) that activates osteoblasts to produce receptor activator of nuclear factor kappa-B ligand (RANKL) and downregulate osteoprotegerin (OPG). This in turn activates osteoclast precursors, leading to osteolysis. The breakdown of the bone matrix releases bone-derived growth factors, such as transforming growth factor-β and insulin-like growth factor 1, and raises extracellular calcium concentrations. The growth factors bind to receptors on the cell surface of the tumor cells and activate SMAD and MAPK signaling, extracellular calcium binds and activates calcium pumps leading to tumor cell proliferation and the production of PTHrP, thereby causing a vicious cycle of events that result in osteolytic lesions and the progression of cancer metastasis (8–10). This theory explained how bone cells and cancer cells could have a reciprocal relationship. However, it does not take into consideration the contribution of the other resident cells of the bone marrow, such as the cells of the immune system, which have also been shown to play an important part in cancer progression (11, 12). In recent years, the contribution of bone marrow adipocytes (BMAs) has also come into question. Cancer primarily occurs in older people whose bone marrow is highly populated by adipocytes. In early childhood, the bone marrow exists in a predominantly red/hematopoietic osteogenic state (13) and there are few if any detectable adipocytes, however, by the age of 25, ~70% of bone marrow volume is filled with adipose tissue (14). This constitutes around 2 kg of marrow adipose tissue in a healthy adult that represents over 10% of total adipose mass (15). Longitudinal analysis of patients from birth to 90 years have demonstrated that even after this initial exponential accumulation, there continues to be a gradual increase in adipocyte number throughout adult life (16). These cells were previously thought to be inert space filling bystanders (17). However, recently that way of thinking has changed and it is becoming clear that adipocytes have a definite and significant contribution to the bone microenvironment and, thus, the establishment of metastatic disease.

## Bone Marrow Adipocytes

Over the last century, our knowledge and understanding of the role of BMAs has been slowly growing. At the beginning of the nineteenth century, it was determined that the skeleton is filled with areas consisting of yellow or red marrow that are distributed in a defined pattern (18). A number of decades later, it was shown using elegant ultrastructural studies that BMAs develop from a unique progenitor when compared to white adipocytes (19), and that there are two distinct populations of BMAs that respond differently to hematopoietic demands (20). This phenomenon was also shown by Scheller and colleagues that marrow adipose tissue may exist in two isoforms, “constitutive” that forms early on in development in sites such as the distal tibia, and “regulated” that tends to develop with aging and is located interspersed with hematopoietic cells at sites such as the proximal femur and lumbar vertebrae (21). The fact that marrow adipose tissue develops in a conserved, spatial, and temporal manner implies that it is the product of a defined developmental event; suggesting that it has a distinct physiological function. Its phenotype resembles both brown and white adipose tissue; and unlike white adipose tissue, it is not strictly associated with BMI or body fat. It is known to be increased in patients suffering from anorexia nervosa, despite their lean appearance (22). It is also elevated in young mice in response to calorie restriction (23). The main functions of bone marrow adipose tissue are to serve as an energy reservoir and secrete fatty acids, cytokines, and adipokines. BMAs store energy in the form of lipid and release triglycerides and fatty acids in response to energy demands. They are smaller than their visceral counterparts; however, due to enhanced triacylglycerol synthesis the net effect of fatty acid uptake is similar (24). BMAs are both an endocrine target and have endocrine-like functions: responding to growth hormones insulin and thyroid hormone, as well as releasing cytokines such as IL-6, IL-1β, and TNF-α (25). They also secrete adipokines, among them leptin and adiponectin, which regulate calorie uptake and insulin sensitivity, respectively. They can potentially influence neighboring cells via autocrine, paracrine, and endocrine signaling (26) making them an influential component of the bone microenvironment. BMAs arise from multipotent mesenchymal progenitors; these cells have the capacity to differentiate into several cell types, including myocytes, chondrocytes, osteoblasts, and adipocytes (27, 28). It is thought that downstream of these multipotent progenitors, bipotent osteoblast–adipocytes progenitors form an intermediate in the process of cell commitment to these two cell lineages (29). These bipotent cells are stimulated to commit to either cell lineage by the presence of adipogenic vs. osteogenic factors within the bone microenvironment that activate their respective transcriptional programs (30, 31). This creates an inverse reciprocal relationship between osteoblastogenesis and adipogenesis, with factors that promote one of these processes usually inhibiting the other. Many have likened it to a see-saw, when one process goes up the other often comes down and vice versa (32). It has also been suggested that mature osteoblasts and adipocytes are able to dedifferentiate and transdifferentiate from one phenotype to the other (33). Work by Martin and colleagues also demonstrated that BMAs are able to influence their environment by secreting extracellular vesicles containing adipogenic mRNA transcripts. These could be taken up and expressed by neighboring osteoblasts, thereby weakening their lineage commitment (34, 35). The net result of this close relationship is that if the balance tips in flavor of adipogenesis, osteoblast number may be reduced, resulting in a decrease in bone, so compromising bone strength. BMAs are not only negative regulators of bone formation but also serve to inhibit hematopoiesis. The number of BMAs has been shown to be inversely correlated with the hematopoietic activity of the marrow (36). The evidence to support the notion that BMAs play a metabolic role in the bone is quite clear; however, the influence they have in the context of cancer development and progression is still poorly understood.

## White Adipose Tissue; A Growing Contributor to Cancer Risk and Progression

**Figure 1:**
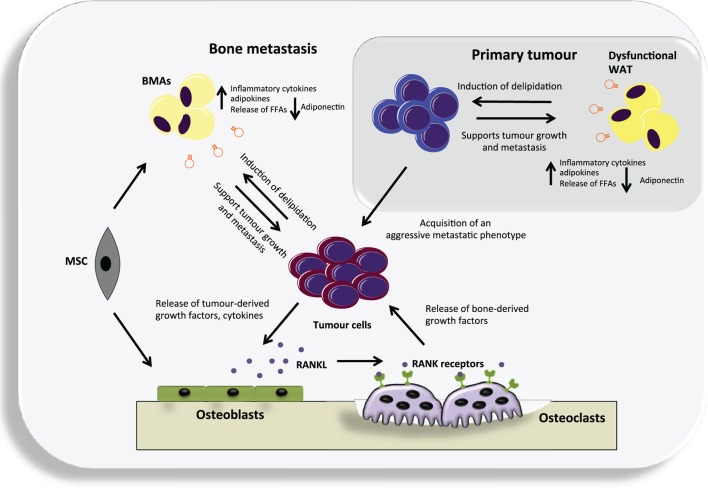
**An overview of the potential contribution of both white adipose tissue (WAT) and bone marrow adipocytes (BMAs) to the vicious cycle of bone metastases**. Dysfunctional WAT releases an increased level of a number of adipokines and pro-inflammatory cytokines that can support tumor growth. In turn, cancer cells may cause delipidation of adipocytes to fuel their growth and the acquisition of an aggressive metastatic phenotype. They can then metastasize to the adipocyte-rich environment of the bone where they may continue to utilize BMAs generated from mesenchymal stem cells (MSC) that reside in the bone, as a source of fuel. Cancer cells residing in the bone produce various cytokines and growth factors that primarily target osteoblasts, causing them to increase their production of receptor activator of nuclear factor kappa-B ligand (RANKL). RANKL in turn targets the RANK receptor expressed by osteoclast precursors and leads to differentiated and activated osteoclasts. The osteoclasts degrade the bone releasing numerous factors. Many of these factors act positively upon the cancer cells resulting in a vicious cycle.

White adipose tissue (WAT) was the first adipose tissue type to be heavily studied in relation to cancer development and progression. In 2002, the International Agency for Research on Cancer (IARC) conducted an evaluation on whether there was a link between weight and cancer. They concluded that the risk of developing some cancers was increased with weight gain (37). Since then, there have been numerous studies investigating the association between increased adiposity and cancer, suggesting that obesity is associated with an increased risk of developing a number of different tumor types, such as colon, breast, and endometrial cancer (38–40). As adipose tissue expands, adipocytes enlarge to store excess energy intake. This causes an increased production of a number of different adipokines and inflammatory cytokines coupled with a decrease in adiponectin, as well as a diminished ability of adipocytes to store surplus-free fatty acids. These changes are associated with dysfunctional WAT that often leads to insulin resistance. In turn, the suppression of lipolysis by insulin is inhibited in insulin resistance, resulting in an increased release of free fatty acids, thereby setting up a vicious cycle of events (40). Lipids are crucial for malignant tumors as they are necessary for the synthesis of membrane constituents; they are also an effective bioenergetic source when metabolic demands are high. In 2011, Nieman et al. demonstrated that adipocytes–ovarian cancer cell co-culture led to the direct transfer of lipids from adipocytes to ovarian cancer cells and promoted *in vitro* and *in vivo* tumor growth. Furthermore, co-culture induced lipolysis in adipocytes and β-oxidation in cancer cells, suggesting that the adipocytes act as an energy source for the cancer cells (41). Adipocytes were also shown to promote the direct migration of prostate cancer cells (42), as well as promote colon cancer proliferation (43). Similar findings have been reported in breast cancer studies, which demonstrate that adipocytes located close to invasive cancer cells are essential for breast tumor development and progression (44). Moreover, adipocytes promote drug resistance in HER2 positive breast cancer cells, suggesting that they bestow a level of protection upon tumor cells (45). Dysfunctional WAT appears to have a tumor-promoting role; it may be that cancer cells that have been in close proximity to adipocytes are primed to settle in an adipocyte-rich secondary site, making the bone a hospitable and permissive environment (Figure 1).

## Bone Marrow Adipocytes; Fueling Cancer Progression

Once WAT had been identified as a driver of cancer progression, the contribution of adipocytes from different anatomical sites came into question. There are now a number of publications highlighting the importance of BMAs as a lipid source that can be utilized by cancer cells to promote proliferation, migration, and invasion (46, 47). A key factor implicated in Nieman’s 2011 study was fatty acid binding protein 4 (FABP4); a lipid chaperone that mediates lipid trafficking and transfer of free fatty acids, predominantly expressed in adipocytes, macrophages, and endothelial cells (41). Herroon et al. took these findings further using a mouse model of diet-induced marrow adiposity and demonstrated that FABP4 along with interleukin 1β and its target gene, oxidative stress protein, heme oxygenase 1 (HMOX-1) was also upregulated in prostate cancer cells that were in direct contact with BMAs (46). Taken together, these studies support the fact that cancer cells are able to utilize marrow adipocyte-supplied lipids to thrive in skeletal sites. They also open up a number of questions as to whether BMAs are utilized in the same manner as WAT or whether they offer different environmental advantages to some cancer types over others.

## Adipokines/Cytokines

Similarly to WAT, BMAs are not only an effective source of energy they also secrete a plethora of bioactive substances, such as IL-1β, IL-6, leptin, adiponectin, VCAM-1, TNF-α, and VEGF (25). As well as being important in the context of maintaining healthy bone, these secreted cytokines, adipokines, and growth factors can also influence cancer cell behavior and survival. Increased IL-1β secretion coupled with increased leptin expression was shown to recruit breast cancer cells to colonize in bone marrow adipose tissue (47). IL-6, TNF-α, CXCL12, and leptin were shown to promote cell proliferation and migration, as well as inhibit apoptosis and activate autophagy to promote chemotherapy resistance in multiple myeloma (25, 48–51). In prostate cancer, the chemokines, CXCL1 and CXCL2, have been implicated in the progression of associated bone disease by activating osteoclastogenesis and thus promoting tumor cell survival (52). Similarly, in melanoma increased IL-6 triggered an increase in osteoclastogenesis resulting in tumor cell proliferation (53). BMAs clearly influence cancer cell establishment and progression in bone. However, they also play a known tumor-suppressive role. BMAs are the largest source of circulating adiponectin in the body, far more than white adipose tissue (54). Adiponectin has been shown to exert anti-tumor effects (55, 56), and its levels are inversely correlated with a number of different cancers, such as myeloma, breast, prostate, colon, and endometrial cancer (57, 58), and are associated with poor prognosis (59). Interestingly, unlike other adipokines, such as leptin, circulating adiponectin levels are also decreased in obesity, which appears to be somewhat paradoxical as the numbers of adipocytes are increased. Reduced circulating adiponectin in obesity is likely derived from dysfunctional adipocytes that have decreased adiponectin expression and secretion, which may be the result of mitochondrial dysfunction or increased inflammation, hypoxia or endoplasmic reticulum stress due to the challenge of a high fat diet (60). These findings trigger the question as to what causes the downregulation of adiponectin expression in cancer? Is it a consequence of cancer progression or a cause? Do cancer cells secrete factors that directly regulate adiponectin levels? Are adiponectin levels compromised before cancer development by conditions, such as obesity, that allow for a more permissive environment? More research needs to be done to address these questions and to validate whether adiponectin signaling may be a future therapeutic target.

## Targeting Bone Marrow Adipocytes

Given the mounting evidence to support the negative role of BMAs in cancer progression, as is further discussed in the context of myeloma by Falyank et al. (61), targeting these cells either alone or in combination with conventional therapeutics may be a promising approach. Due to the ability of cancer cells to induce lipidation of BMAs to support their energy expenditure, drugs are now being developed that target essential molecules of fatty acid synthesis and uptake. Chemical or RNAi mediated inhibition of fatty acid synthase, acetyl-CoA-carboxylase or ATP-citrate lyase was shown to attenuate cancer cell proliferation and cell survival in both *in vitro* and *in vivo* models (62–64). Another approach is to regulate the balance between osteogenesis and adipogenesis by targeting PPARγ or glucocorticoid receptor signaling, thereby preventing an increase in marrow adiposity (65). The modulation of adipokines although still in its infancy has also shown encouraging effects. Leptin receptor antagonists were shown to inhibit tumor cell growth in a model of triple-negative breast cancer (66). Enhancement of circulating adiponectin levels by the apolipoprotein mimetic L-4F caused cancer cell death in a mouse model of multiple myeloma (55). Furthermore, a peptide-based adiponectin receptor agonist ADP 355 was shown to suppress the growth of orthotopic human breast cancer xenografts by ~31% (67). As we begin to gain more knowledge of the exact contribution of BMAs to disease progression, targeting strategies will inevitably become clearer. First, though we need to understand whether BMAs are simply power stations that fuel an inevitable process? If they were removed from the equation, would the cancer cells still home to the bone and thrive? Or are BMAs an integral part of the metastatic process, fertilizing the soil so circulating cancer cells can settle and progress. Interestingly, marrow adipose tissue unlike white adipose tissue increases in times of starvation. Calorie restriction has been show to not only increase marrow adiposity but also extend lifespan in a number of diverse species, such as rodents and primates (68, 69), and is associated with a decrease in cancer risk (70). These findings suggest that BMAs have a protective function that is contradictory to the evidence to suggest that they promote cancer progression. However, one question that remains unresolved is whether the BMAs generated by weight gain and sedentary behavior are the same as those generated by calorie restriction? It may be that BMAs generated by calorie restriction are more metabolically aware and, therefore, less responsive to cancer cell manipulation. However more research needs to be done to explore these possibilities. Targeting BMAs as part of a combination therapy may prove to be a valuable tool; however, first there needs to be a greater understanding of the balance between the tumor-promoting and tumor-suppressive effects of these cells.

## Conclusion

As the field of marrow adiposity advances and we come to fully appreciate and understand the influence BMAs have on disease progression, drugs that target these cells may start to come into their own and open up new therapeutic avenues. These advances will hopefully move us one step closer to successfully treating lethal metastatic cancer.

## Author Contributions

All authors listed have made substantial, direct, and intellectual contribution to the work and approved it for publication.

## Conflict of Interest Statement

The authors declare that the research was conducted in the absence of any commercial or financial relationships that could be construed as a potential conflict of interest.
